# Simulation as a preoperative planning approach in advanced heart failure patients. A retrospective clinical analysis

**DOI:** 10.1186/s12938-018-0491-7

**Published:** 2018-05-02

**Authors:** Massimo Capoccia, Silvia Marconi, Sanjeet Avtaar Singh, Domenico M. Pisanelli, Claudio De Lazzari

**Affiliations:** 1 Department of Cardiac Surgery, University Hospitals of Leicester NHS Trust, Leicester, UK; 2 Department of Biomedical Engineering, University of Strathclyde, Glasgow, UK; 3 National Research Council, Institute of Clinical Physiology, Rome, Italy; 4 Golden Jubilee National Hospital, Clydebank, Glasgow UK; 5 National Research Council, Institute of Cognitive Sciences and Technologies, Rome, Italy; 6 National Institute for Cardiovascular Research (I.N.R.C.), Bologna, Italy

**Keywords:** Ventricular assist device, Heart failure, Simulation, CARDIOSIM^©^, Modelling

## Abstract

**Background:**

Modelling and simulation may become clinically applicable tools for detailed evaluation of the cardiovascular system and clinical decision-making to guide therapeutic intervention. Models based on pressure–volume relationship and zero-dimensional representation of the cardiovascular system may be a suitable choice given their simplicity and versatility. This approach has great potential for application in heart failure where the impact of left ventricular assist devices has played a significant role as a bridge to transplant and more recently as a long-term solution for non eligible candidates.

**Results:**

We sought to investigate the value of simulation in the context of three heart failure patients with a view to predict or guide further management. CARDIOSIM^©^ was the software used for this purpose. The study was based on retrospective analysis of haemodynamic data previously discussed at a multidisciplinary meeting. The outcome of the simulations addressed the value of a more quantitative approach in the clinical decision process.

**Conclusions:**

Although previous experience, co-morbidities and the risk of potentially fatal complications play a role in clinical decision-making, patient-specific modelling may become a daily approach for selection and optimisation of device-based treatment for heart failure patients. Willingness to adopt this integrated approach may be the key to further progress.

## Background

Physiological fluid flow modelling using computational fluid dynamics (CFD) has increased our understanding of complex system interactions with particular reference to problem solving in a clinical environment [1–6]. Functional analysis and assessment of the cardiovascular system through computational modelling based on imaging techniques has certainly received great attention in relation to its diagnostic value and planning approach [7–11].

Mathematical modelling and simulation may become clinically applicable tools for detailed evaluation of the cardiovascular system and clinical decision-making to guide therapeutic intervention [12]. Models based on pressure–volume relationship and lumped-parameter representation of the cardiovascular system may be a suitable choice given their simplicity and versatility [13–19]. Although they provide less detailed predictions of pressure and flow waveforms, these models have shown great flexibility in simulating the haemodynamics of different cardiovascular conditions and therapeutic interventions with the potential to be run in real time on desktop, laptop or mobile devices [12]. The successful clinical application of this approach requires further haemodynamics teaching to the medical community although its importance is still far from being fully appreciated [19–21].

This approach has great potential for application in heart failure where the impact of left ventricular assist devices (LVADs) has played a significant role as a bridge to transplant and more recently as a long-term solution for non eligible candidates. Continuous flow rotary blood pumps are currently the most popular devices because of their smaller size, increased reliability and higher durability compared to pulsatile-flow devices. The trend towards their use is increasing. Mathematical modelling and computer simulation are invaluable tools to investigate the interactions between LVADs and the cardiovascular system [22–24]. CFD simulations for continuous flow LVADs are usually performed under steady flow conditions where the inlet boundary is set to a specific steady velocity profile while the outlet boundary is fixed at a steady pressure. The actual unsteady boundary conditions will be dependent on the flow from the heart and the aortic pressure, which are the result of the interactions between the device and the cardiovascular system [25]. The study of the interaction between LVADs and the whole cardiovascular system with a 3-D CFD model is highly demanding although specific parts of the assisted circulation have been developed with this method [26] but their practical application may be limited at present. A more simplified approach is based on one-dimensional (1-D) or lumped parameter (0-D) models where space dependence is either confined to the axial coordinate (1-D) or addressed by splitting the cardiovascular system in compartments (0-D) [27].

The native ventricular behaviour can be modelled according to the time-varying elastance theory [28–30], which remains a landmark despite its limitations [31] and previous criticism when applied to a mechanically supported left ventricle [32]. Significant elastance changes are observed during circulatory support with a blood displacement pump because of extreme and fast changing loading conditions. Therefore, the relationship between elastance and contractility may be no longer applicable when a second pump is connected to the systemic circulation [32]. Elastance changes are also observed with continuous flow pumps where an increasing pump flow is related to a constant end-systolic volume, a decrease in end-diastolic volume and a maximum left ventricular pressure increase with a gradual increase in the slope (*Ees*) of the end-systolic pressure–volume relationship (ESPVR) to justify dissociation between contractility and elastance [32]. Although a linear model has been proven sufficiently accurate [33–35] and adequate for realistic simulations of the instantaneous pressure–volume relation [36], recent multi-scale modelling of the cardiovascular system [37] based on previously developed approaches [38–40] has successfully addressed the limitations of the time-varying elastance theory with particular reference to load-dependence of ESPVR. Further modelling techniques can describe a failing cardiovascular system [41] and ventricular interactions [42–44]. To address the shortcomings of the original time-varying elastance theory, a nonlinear time-varying lumped parameter model of the cardiovascular system [45] can be modified to include the inter-ventricular septum and a rotor-dynamic, continuous flow LVAD [46]. This is a more accurate heart failure model where the ESPVR is a unimodal function that takes into account the descending limb of the Frank–Starling curve, making it particularly suitable to study ventricular interactions and the leftward septal shift secondary to left ventricular decompression following LVAD insertion. Outlet tube pump modelling with a linearly flow-dependent resistance is also used to study pulsatile and continuous flow LVADs. The resistance consists of flow-proportional and constant components in the context of a steady state environment but dynamic modelling with a time-varying resistance has been considered more recently [47, 48]. A Lagrange multiplier coupling approach [49] can be applied to LVAD modelling [50] using fictitious domain methods [51, 52] to address the interactions between the LVAD cannula and the ventricle at the expense of significant increase in computational time and instability in specific regions of interface between the fluid and solid meshes. To overcome these limitations, a fluid–solid left ventricular model coupled with a 0-D Windkessel model [53, 54] already successfully applied to different types of LVAD [55–57] can be used where optimization with high order interpolation at the fluid–solid boundary allows simulations of fluid–solid interaction over a complete cardiac cycle during LVAD support [58].

Following these considerations, we sought to investigate the value of simulation in the context of three heart failure patients previously discussed at a multidisciplinary meeting with a view to predict or guide further management. The aim was to compare the outcome of the simulations with the previously made clinical decision in order to find out any relationship that may be applicable on a routine basis in future patient assessment. The key elements would be:

- A more targeted approach for different group of patients;
- More quantitative evaluation in the clinical decision process;
- The predictive value of simulation;
- Preoperative planning and treatment optimisation.

## Methods

We retrospectively analysed the haemodynamic data of three heart failure patients previously discussed at a multidisciplinary meeting and subsequently treated accordingly. Given the retrospective nature of this study, informed consent was waived [59]. The aim was to reproduce the preoperative haemodynamic status of these patients and then carry out simulations in the presence of a ventricular assist device in order to evaluate their suitability for prolonged mechanical support or other intervention.

The study was carried out using CARDIOSIM^©^, which is a software package developed by the Cardiovascular Numerical Modelling LAB linked to the Institute of Clinical Physiology, CNR, Rome, Italy [60]. This is a numerical simulator of the cardiovascular system based on lumped parameter models, modified time-varying elastance and pressure–volume analysis of ventricular function. The software is interactive and capable of reproducing physiological and pathological conditions for clinical decision-making in a controlled environment [14, 15]. The main feature is a modular approach with an updatable library of numerical models of different sections of the cardiovascular system, which can be assembled according to the need of the simulation. The software is particularly suitable to study the interactions with pulsatile or continuous flow ventricular assist devices [15, 22–24], intra-aortic balloon pump, artificial lung, biventricular assist device and biventricular pacing [15, 18, 61–63].

When the native atrial and ventricular behaviour are modelled, the numerical simulator allows choosing between two different modules [16–19] based on the time-varying elastance theory. For the purpose of our simulations, we have considered the module where left and right ventricular elastances $e_{lv} \left( t \right)$ and $e_{rv} \left( t \right)$ are described as a function of the characteristic elastance in systole ($E_{lv,s}$ and $E_{rv,s}$) and in diastole ($E_{lv,d}$ and $E_{rv,d}$), and an activation function ($\overline{e}_{lv} \left( t \right)$ and $\overline{e}_{rv} \left( t \right)$) as follows [54]:

$$ \begin{aligned} e_{lv} (t) & = E_{lv,d} + \left[ {\frac{{E_{lv,s} - E_{lv,d} }}{2}} \right] \cdot \overline{e}_{lv} (t) \\ e_{rv} (t) & = E_{rv,d} + \left[ {\frac{{E_{rv,s} - \, E_{rv,d} }}{2}} \right] \cdot \overline{e}_{rv} (t) \\ \end{aligned} $$

where

$$ \overline{\text{e}}_{\text{lv}} ( {\text{t) }}= \;\overline{\text{e}}_{\text{rv}} ( {\text{t)}} = \left\{ {\begin{array}{*{20}l} { 1- { \cos }\left( {\frac{\text{t}}{{{\text{T}}_{\text{T}} }}\pi } \right)\;\begin{array}{*{20}l} {} & {} & {} & {0 \le t \le T_{T} } \\ \end{array} } \\ {1 + \cos \left( {\frac{{t - T_{T} }}{{T_{TE} - T_{T} }}\pi } \right)\begin{array}{*{20}l} {} & {T_{T} < t \le T_{TE} } \\ \end{array} } \\ {0\begin{array}{*{20}l} {} & {} & {} & {\begin{array}{*{20}l} {\begin{array}{*{20}l} {} & {} \\ \end{array} } & {} & & {} & & {} & {} & {T_{TE} < t \le T} \\ \end{array} } \\ \end{array} } \\ \end{array} } \right. $$

$T$ is the duration of the ECG signal (heart period), $T_{TE}$ is the end of ventricular systole and $T_{T}$ is the T-wave peak time [15, 19].

Instantaneous left and right ventricular pressures $P_{lv} \left( t \right)$ and $P_{rv} \left( t \right)$ are obtained from the instantaneous ventricular free wall volumes and elastances as follows [54]:

1 $$ \begin{aligned} {\text{P}}_{\text{lv}} ( {\text{t)}}\; & { = }\;{\text{P}}_{\text{lv,0}} {\text{ + e}}_{\text{lv}} ( {\text{t)}} \cdot \left[ {{\text{V}}_{lv}^{*} ( {\text{t) }} - {\text{V}}_{\text{lv,0}} } \right] \\ {\text{P}}_{\text{rv}} ( {\text{t)}}\; & { = }\;{\text{P}}_{\text{rv,0}} {\text{ + e}}_{\text{rv}} ( {\text{t)}} \cdot \left[ {{\text{V}}_{rv}^{*} ( {\text{t)}} - {\text{V}}_{\text{rv,0}} } \right] \\ \end{aligned} $$

where $V_{lv}^{*} \left( t \right)$ and $V_{rv}^{*} \left( t \right)$ are the instantaneous left and right ventricular free wall volumes; $V_{lv, 0}$ and $V_{rv, 0}$ are the resting left and right ventricular volumes; $P_{lv, 0}$ and $P_{rv, 0}$ are the resting left and right ventricular pressures.

The same approach is applicable to the atrial chambers, the interventricular (IVS) and interatrial (IAS) septum [15]. The IVS is the key factor where the properties of one ventricle are a function of the contra-lateral one [64]. When the time-varying elastance theory is applied, from Eq. (1) we obtain [15, 19]:

2 $$ \begin{aligned} P_{lv} (t) & = \left[ {\frac{{e_{Vsp} (t) \cdot e_{lv} (t)}}{{e_{lv} (t) + e_{Vsp} (t)}}} \right] \cdot \left[ {V_{lv} (t) - V_{lv,0} } \right] + \left[ {\frac{{e_{lv} (t)}}{{e_{lv} (t) + e_{Vsp} (t)}}} \right] \cdot P_{rv} (t) + \left[ {\frac{{e_{Vsp} (t)}}{{e_{lv} (t) + e_{Vsp} (t)}}} \right] \cdot P_{lv,0} \\ P_{rv} (t) & = \left[ {\frac{{e_{Vsp} (t) \cdot e_{rv} (t)}}{{e_{Vsp} (t) + e_{rv} (t)}}} \right] \cdot \left[ {V_{rv} (t) - V_{rv,0} } \right] + \left[ {\frac{{e_{rv} (t)}}{{e_{Vsp} (t) + e_{rv} (t)}}} \right] \cdot P_{lv} (t) + \left[ {\frac{{e_{Vsp} (t)}}{{e_{Vsp} (t) + e_{rv} (t)}}} \right] \cdot P_{rv,0} \\ \end{aligned} $$

where $e_{Vsp} \left( t \right)$ is the septal ventricular elastance described in [15, 19]. $V_{lv} \left( t \right)$ and $V_{rv} \left( t \right)$ represent the instantaneous left and right ventricular volume respectively.

Equation (2) are essential for the model to simulate ventricular interactions with or without mechanical circulatory support.

Similarly, the following equations were used to reproduce the atrial behaviour [15, 19]:

3 $$ \begin{aligned} P_{la} (t) &= \left[ {\frac{{e_{Asp} (t) \cdot e_{la} (t)}}{{e_{la} (t) + e_{Asp} (t)}}} \right] \cdot \left[ {V_{la} (t) - V_{la,0} } \right] + \left[ {\frac{{e_{la} (t)}}{{e_{la} (t) + e_{Asp} (t)}}} \right] \cdot P_{ra} (t) + \left[ {\frac{{e_{Asp} (t)}}{{e_{la} (t) + e_{Asp} (t)}}} \right] \cdot P_{la,0} \hfill \\ P_{ra} (t) &= \left[ {\frac{{e_{Asp} (t) \cdot e_{ra} (t)}}{{e_{Asp} (t) + e_{ra} (t)}}} \right] \cdot \left[ {V_{ra} (t) - V_{ra,0} } \right] + \left[ {\frac{{e_{ra} (t)}}{{e_{Asp} (t) + e_{ra} (t)}}} \right] \cdot P_{la} (t) + \left[ {\frac{{e_{Asp} (t)}}{{e_{Asp} (t) + e_{ra} (t)}}} \right] \cdot P_{ra,0} \hfill \\ \end{aligned} $$

where $e_{Asp} \left( t \right)$ is the septal atrial elastance described in [15, 19]; $e_{la} \left( t \right)$ and $e_{ra} \left( t \right)$ are the left and right atrial elastances; $P_{la} \left( t \right)$ [$V_{la} \left( t \right)$] and $P_{ra} \left( t \right)$ [$V_{ra} \left( t \right)$] are the instantaneous left and right atrial pressures [volumes]; $V_{la, 0}$ and $V_{ra, 0}$ are the resting left and right atrial volumes.

Equation (3) are essential for the model to simulate atrial interactions with or without mechanical circulatory support.

**Fig. 1:**
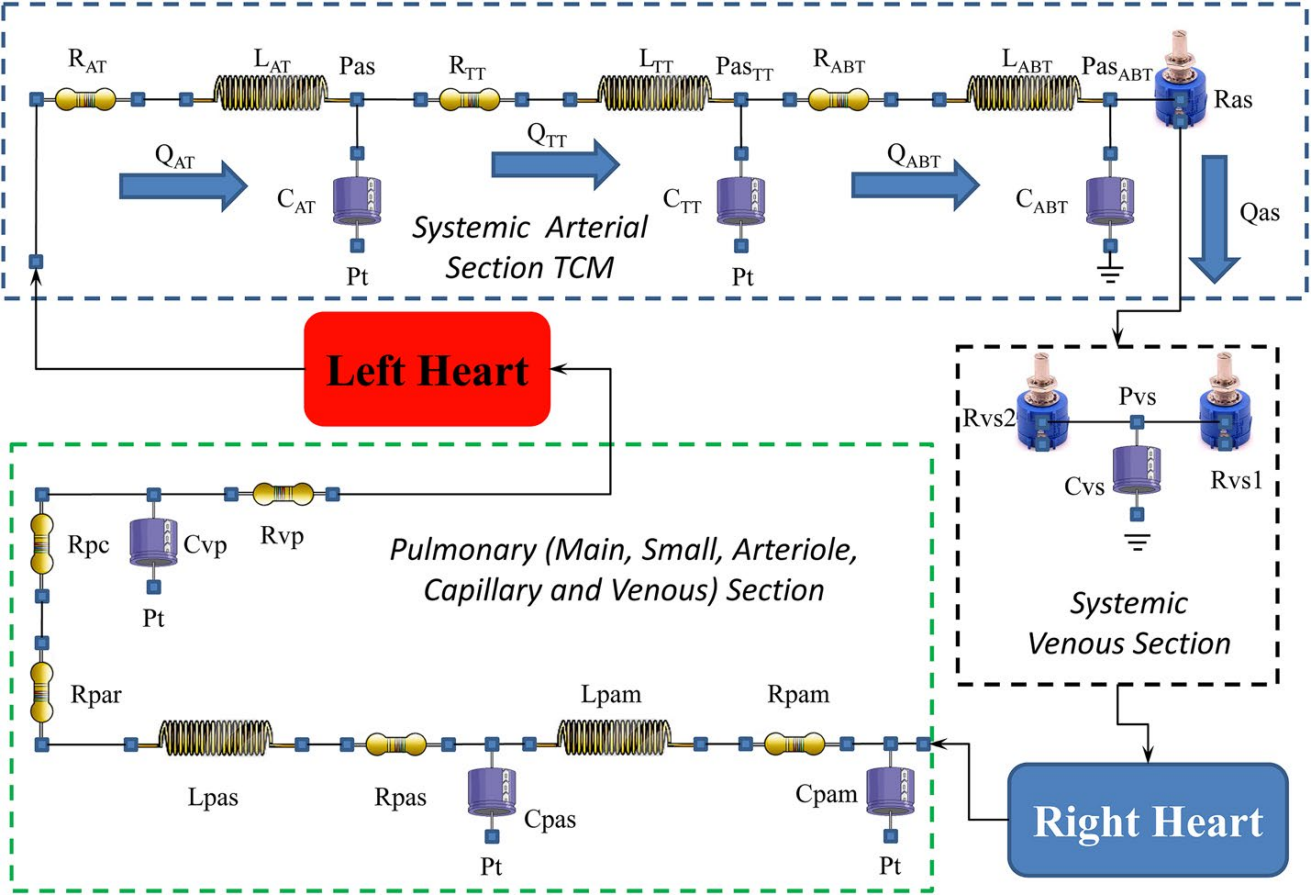
Electrical analogue model of the cardiovascular system. The systemic arterial section consists of three RLC elements representing the aortic (R_AT_, L_AT_ and C_AT_), thoracic (R_TT_, L_TT_ and C_TT_) and abdominal (R_ABT_, L_ABT_ and C_ABT_) tract respectively. Ras is the variable systemic peripheral resistance. The systemic venous section consists of two variable resistances (Rvs1 and Rvs2) and a compliance (Cvs). The main (small) pulmonary section is reproduced by a RLC element: Rpam, Lpam and Cpam (Rpas, Lpas and Cpas). The arteriole (capillary) bed behaviour is reproduced by a single resistance Rpar (Rpc). The pulmonary venous section consists of a compliance (Cvp) and a resistance (Rvp). Pt is the mean intrathoracic pressure

The systemic arterial section consists of the aortic, thoracic and abdominal compartment and is modelled as described in [14, 65] with RLC circuits (Fig. 1). The whole venous system is modelled with only one RC circuit. The inertial forces have been neglected. The pulmonary arterial section consists of the main pulmonary artery and the intra-pulmonary vascular bed, each modelled with a RLC lumped element. The pulmonary arteriole compartment consists of a single resistance in view of their relative stiffness and the fact that inertial forces are negligible. The pulmonary capillary section is also modelled with a single resistance as previously reported [18]. Coupling between the ventricles and the circulation is obtained by using ideal valves [17, 22]. The potential for research, clinical application and training is significant and well documented [14, 15, 21, 66].

**Fig. 2:**
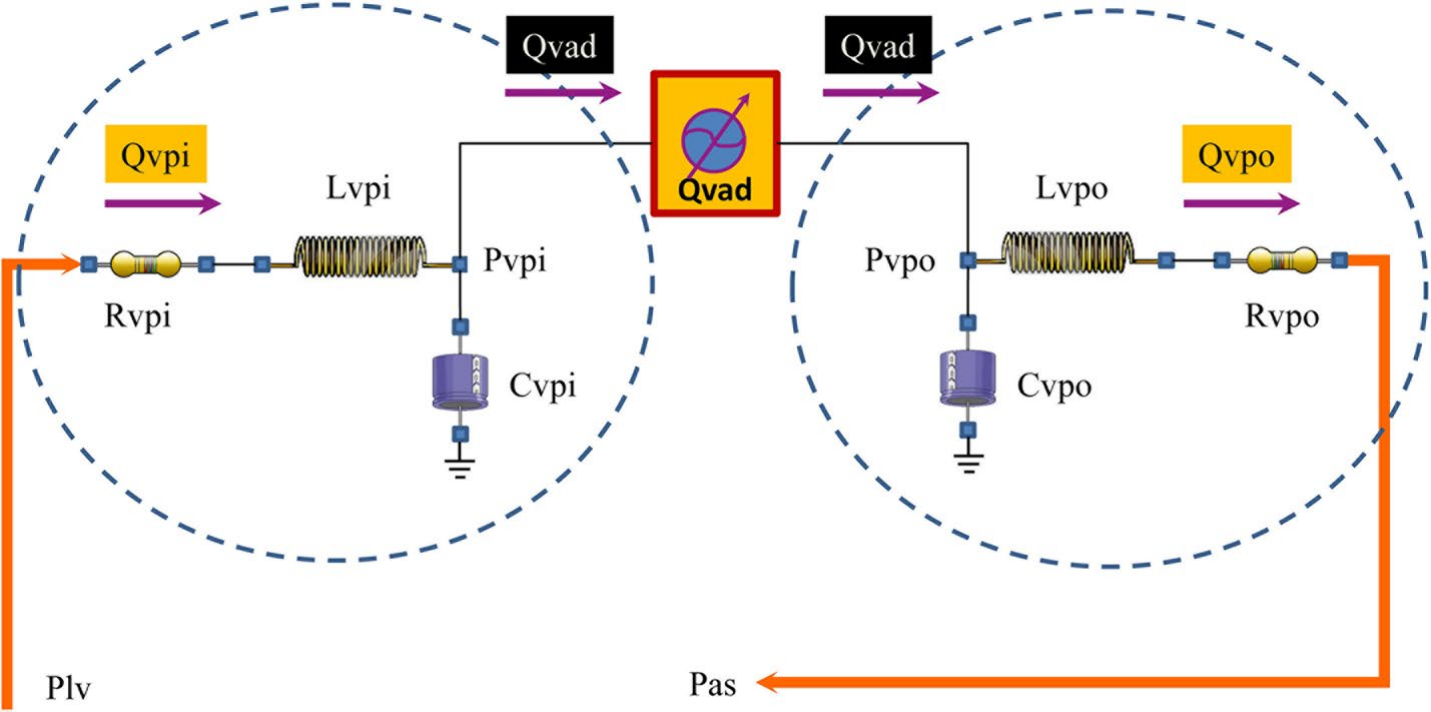
Electrical analogue model of the Berlin Heart INCOR pump. Plv ad Pas are the left ventricular and systemic arterial pressures respectively. Input (output) pump cannula is modelled with a resistance Rvpi (Rvpo), a compliance Cvpi (Cvpo) and an inertance Lvpi (Lvpo). Qvad is the pump flow and Qvpi (Qvpo) is the input (output) cannula flow

Figure 2 shows the electric analogue of the LVAD model (Berlin Heart INCOR Pump) [55] integrated in the software and used for our simulations. The inlet and outlet cannulae are modelled with RLC elements $R_{vpi} , L_{vpi} , C_{vpi}$ and $R_{vpo} , L_{vpo} , C_{vpo}$.

The inlet and outlet cannulae flows $Q_{vpi}$ and $Q_{vpo}$ are calculated as follows:

$$ \left\{ \begin{array} {l}\left( {P_{lv} + P_{t} } \right) - P_{vpi} = Q_{vpi} \cdot R_{vpi} + L_{vpi} \frac{{dQ_{vpi} }}{dt} \hfill \\ Q_{vpi} = Q_{vad} + C_{vpi} \frac{{dP_{vpi} }}{dt} \hfill \\ \end{array} \right.\begin{array}{*{20}c} {} & {} & {} \\ \end{array} \left\{ \begin{array} {l} P_{vpo} - \left( {P_{as} + P_{t} } \right) = Q_{vpo} \cdot R_{vpo} + L_{vpo} \frac{{dQ_{vpo} }}{dt} \hfill \\ Q_{vpo} = Q_{vad} - C_{vpo} \frac{{dP_{vpo} }}{dt} \hfill \\ \end{array} \right. $$

where $P_{t}$ is the mean intrathoracic pressure, $P_{as}$ is the systemic atrial pressure, $P_{vpi}$ and $P_{vpo}$ are the inlet and outlet cannulae pressures.

The LVAD flow $Q_{vad}$ is described by:

$$ \begin{aligned} Q_{vad} & = K_{vad,0} + \omega \cdot K_{vad,1} + K_{vad,2} \cdot \left( {P_{vpo} - P_{vpi} } \right) + K_{vad,3} \cdot \omega \cdot \left( {P_{vpo} - P_{vpi} } \right) \\ & \quad + \;K_{vad,4} \cdot \omega^{2} \cdot \left( {P_{vpo} - P_{vpi} } \right) + K_{vad,5} \cdot \omega \cdot \left( {P_{vpo} - P_{vpi} } \right)^{2} + K_{vad,6} \cdot \omega^{2} \cdot \left( {P_{vpo} - P_{vpi} } \right)^{2} \\ \end{aligned} $$

where

$$ \omega \left( t \right) = A_{0} + A_{p} \cdot \;\sin \left( {\frac{2\pi t}{T} + \varepsilon_{0} } \right) $$

$A_{0}$ is the component of the LVAD speed; $A_{p}$ is the amplitude of the pulsation component; $\varepsilon_{0}$ is the phase difference between the LVAD pulsation component and the native cardiac timing. The setting parameters are listed in Table 1.

**Table 1:** Setting parameters of the LVAD used for the simulations

Parameter	Value	Unit
Inlet and outlet cannulae parameters		
$C_{vpi}$ [$C_{vpo}$]	0.1 [0.1]	mmHg^−1^ ml
$R_{vpi}$ [$R_{vpo}$]	0.01 [0.01]	mmHg s/ml
$L_{vpi}$ [$L_{vpo}$]	1.2 × 10^−4^ [1.2 × 10^−4^]	mmHg s^2^/ml
LVAD parameter		
$K_{vad,1}$	− 3.0361 × 10^−3^	l/min/rpm
$K_{vad,2}$	− 1.23045	l/min/mmHg
$K_{vad,3}$	5.78974 × 10^−4^	l/min/rpm/mmHg
$K_{vad,4}$	− 5.8777 × 10^−8^	l/min/rpm^2^/mmHg
$K_{vad,5}$	− 1.27359 × 10^−6^	l/min/rpm/mmHg^2^
$K_{vad,6}$	2.04834 × 10^−10^	l/min/rpm^2^/mmHg^2^

The first order ordinary differential equations of the cardiovascular model were initially solved using both Euler’s and fourth order Runge–Kutta’s methods. The two numerical methods gave the same results under steady state conditions. The Euler’s method with a time step of 1 ms was preferred because of its lower computational cost compared to the Runge–Kutta’s method. The simulation software was written using Visual Basic language. Statistical analysis was carried out using the unpaired “t” test and correlation coefficients were calculated.

The retrospective analysis was performed on the haemodynamic data of the following patients:

### Patient 1

A 34-year-old patient who sustained an extensive anterior wall myocardial infarction, which was initially treated with a percutaneous interventional procedure and the insertion of a drug eluting stent to the left anterior descending coronary artery. Subsequently, stent occlusion and residual severe left ventricular systolic dysfunction with an ejection fraction of 27% required full anti-failure treatment. Further deterioration required dobutamine infusion and close monitoring. The presence of co-morbidities, particularly high body mass index (BMI), made this patient unsuitable for transplant while the insertion of a left ventricular assist device remained debatable and unlikely to be beneficial. Haemodynamic data from repeated right heart catheter showed persistent elevated pulmonary artery pressures and resistance with significantly reduced right ventricular stroke work index, which increased the need for right ventricular support following LVAD insertion with potential for prolonged intensive care need and increased risk and complications. The final decision following a multidisciplinary team (MDT) meeting was to continue with medical management and palliative treatment. Table 2 shows measured haemodynamic data following right heart catheterisation (RHC) on admission and 4 days later. End-diastolic ventricular volume [EDV = (CO/HR)/EF], end-systolic ventricular volume [ESV = EDV − (CO/HR)] and the slope (*Ea*) of arterial elastance (*Ea *≈ BP_mean_/SV) [65], where SV is the stroke volume, were estimated from the measured parameters.

**Table 2:** Measurements following right heart catheterisation on admission (RHC 1) and after 4 days (RHC 2)

Patient #1	RHC 1 (admission)			RHC 2 (after 4 days)		
	Max	Min	Mean	Max	Min	Mean
BP (mmHg)	85–90	59	69.3	85–90	60	70
RA (mmHg)	35	17	29	38	22	32
RV (mmHg)	61	14	38	71	11	44
PA (mmHg)	62	30	42	70	38	50
PCWP (mmHg)	36	31	32	35	25	34
TPG (mmHg)	10			16		
CO (l/min)	2.7			2.8		
CI (l/min/m^2^)	1.36			1.4		
PVR (wood unit)	3.7			5.7		
RVSWI (g/m^2^/beat)	2.4			2.4		
HR (bpm)	100			95		
BSA (m^2^)	1.98			1.98		
EF_Left_ (%)	27			27		
*Estimated values*						
EDV (ml)	~ 100			~ 109		
ESV (ml)	~ 73			~ 80		
Ea (mmHg/ml)	~ 3.0			~ 2.8		

*BP* blood pressure, *RA* right atrial pressure, *RV* right ventricular pressure, *PA* pulmonary arterial pressure, *PCWP* pulmonary capillary wedge pressure, *TPG* trans-pulmonary pressure gradient, *CO* cardiac output, *CI* cardiac index, *PVR* pulmonary vascular resistance, *RVSWI* right ventricular stroke work index, *HR* heart rate, *BSA* body surface area, *EF*_*Left*_ left ventricular ejection fraction, *EDV* end-diastolic volume, *ESV* end-systolic volume, *Ea* arterial elastance

### Patient 2

A 55-year-old patient with hypertrophic cardiomyopathy (NYH7 mutation), who previously underwent aortic valve replacement with a mechanical prosthesis and subsequently percutaneous intervention to the left anterior descending coronary artery. Left ventricular ejection fraction EF_Left_ was 45% in the context of chronic atrial fibrillation, previous ventricular arrhythmias and renal impairment. Symptoms deterioration required multiple hospital admissions with readjustment of anti-failure treatment and finally commenced on Milrinone (PDE3 inhibitor) infusion. A MDT meeting considered a LVAD not a suitable option and therefore this patient was placed on the transplant list. Table 3 shows measured and estimated haemodynamic data on admission, 1 and 2 months later.

**Table 3:** Measurements following right heart catheterisation on admission (RHC 1), after 1 month (RHC 2) and after 2 months (RHC 3)

Patient #2	RHC 1 (admission)			RHC 2 (after 1 month)			RHC 3 (after 2 months)		
	Max	Min	Mean	Max	Min	Mean	Max	Min	Mean
BP (mmHg)	95–100	58	72	–	–	–	–	–	–
RA (mmHg)	14	2	9	10	4	6	11	1	7
RV (mmHg)	39	2	17	37	1	15	32	-2	14
PA (mmHg)	40	17	27	34	15	26	31	14	22
PCWP (mmHg)	28	7	18	26	8	15	26	11	15
TPG (mmHg)	9			11			7		
CO (l/min)	5.3			7.1			5.6		
CI (l/min/m^2^)	2.26			3.02			2.4		
PVR (wood unit)	1.7			1.55			1.25		
RVSWI (g/m^2^/beat)	8.5			9.35			6.2		
HR (bpm)	65			88			78		
BSA (m^2^)	2.35			2.35			2.35		
EF_Left_ (%)	45			–			–		
*Estimated values*									
EDV (ml)	~ 181			–			–		
ESV (ml)	~ 99			–			–		
Ea (mmHg/ml)	~ 1.1			–			–		

*BP* blood pressure, *RA* right atrial pressure, *RV* right ventricular pressure, *PA* pulmonary arterial pressure, *PCWP* pulmonary capillary wedge pressure, *TPG* trans-pulmonary pressure gradient, *CO* cardiac output, *CI* cardiac index, *PVR* pulmonary vascular resistance, *RVSWI* right ventricular stroke work index, *HR* heart rate, *BSA* body surface area, *EF*_*Left*_ left ventricular ejection fraction, *EDV* end-diastolic volume, *ESV* end-systolic volume, *Ea* arterial elastance

### Patient 3

A 52-year-old patient who previously sustained a myocardial infarction requiring coronary artery bypass grafting and subsequently implantable cardioverter defibrillator (ICD) insertion because of ventricular arrhythmias. Significant deterioration of the clinical picture had already required multiple hospital admission in a background of dilated left ventricle (left ventricular end-diastolic diameter 8.1 cm) with severe systolic (left ventricular ejection fraction 15%) and diastolic dysfunction (E/A ratio 3.4) and severe pulmonary hypertension. Finally, Milrinone and diuretic infusion were commenced and an intra-aortic balloon pump was inserted. Following a MDT meeting, this patient was listed for transplant with a view to LVAD insertion if further deterioration occurred. Table 4 shows measured and estimated haemodynamic data on admission, 15 and 22 days later.

**Table 4:** Measurements following right heart catheterisation on admission (RHC 1), after 15 days (RHC 2) and after 22 days (RHC 3)

Patient #3	RHC 1 (admission)			RHC 2 (after 15 days)			RHC 3 (after 22 days)		
	Max	Min	Mean	Max	Min	Mean	Max	Min	Mean
BP (mmHg)	100	60	73.3	–	–	–	–	–	–
RA (mmHg)	14	12	9	18	11	15	14	6	10
RV (mmHg)	53	5	–	60	4	28	67	-3	28
PA (mmHg)	58	27	37	75	33	44	75	36	48
PCWP (mmHg)	39	29	31	48	26	35	47	26	33
TPG (mmHg)	6			9			15		
CO (l/min)	4.2			4.5			2.6		
CI (l/min/m^2^)	1.94			2.1			1.2		
PVR (wood unit)	1.4			2			6.8		
RVSWI (g/m^2^/beat)	9.87			11.4			9.15		
HR (bpm)	75			72			68		
BSA (m^2^)	2.16			2.16			2.16		
EF_Left_ (%)	15			–			–		
*Estimated values*									
EDV (ml)	~ 373			–			–		
ESV (ml)	~ 317			–			–		
Ea (mmHg/ml)	~ 1.6			–			–		

*BP* blood pressure, *RA* right atrial pressure, *RV* right ventricular pressure, *PA* pulmonary arterial pressure, *PCWP* pulmonary capillary wedge pressure, *TPG* trans-pulmonary pressure gradient, *CO* cardiac output, *CI* cardiac index, *PVR* pulmonary vascular resistance, *RVSWI* right ventricular stroke work index, *HR* heart rate, *BSA* body surface area, *EF*_*Left*_ left ventricular ejection fraction, *EDV* end-diastolic volume, *ESV* end-systolic volume, *Ea* arterial elastance

The diseased status for each patient was reproduced starting from the measured parameters. Subsequently, LVAD support without and with drug administration (Milrinone) was applied to each patient. The effects induced by Milrinone administration were simulated with the aim to increase heart contractility by 10% and reduce pulmonary and systemic resistances by 10%.

## Results

Table 5 shows (for the first patient) the comparison between measured (second column) and simulated parameters (third column) for “Admission” conditions. In simulated conditions, the simulator calculates the slope *Ees* of the end-systolic pressure–volume relationships (ESPVR) and the ratio *Ea/Ees*. In normal conditions, *Ea/Ees* ratio represents a reliable index of ventricular–arterial coupling. The estimated and simulated *Ea* are similar. The last column of the table shows simulated values during LVAD assistance. EDV, ESV and SV decreased with a leftward shift of the left ventricular loop (in the pressure–volume plane) during LVAD support. Moreover, *Ea* decreased from 3.2 (mmHg/ml) to 2.6 (mmHg/ml) and the *Ea/Ees* ratio decreased from 3.64 to 2.95 during assistance. Two different values for PVR are reported in Table 5: the first one is calculated as the ratio between TPG and left ventricular output flow (CO_VENTR_), the second one is calculated as the ratio between TPG and LVAD flow (Q_VAD_).

**Table 5:** Comparison between measured and simulated parameters for patient 1 on admission

Patient #1	Measured (RHC 1)			Simulation (RHC 1)			LVAD (simulation)		
	Max	Min	Mean	Max	Min	Mean	Max	Min	Mean
BP (mmHg)	85–90	59	69.3	87.2	60.7	69.3	75.2	62.9	68.2
RA (mmHg)	35	17	29	10.5	3.5	6.5	10.3	3.5	6.5
RV (mmHg)	61	14	38	44.2	8.0	20.4	43.5	8.0	20.1
PA (mmHg)	62.0	30.0	42.0	44.0	39.7	42.0	43.3	38.7	40.9
PCWP (mmHg)	36.0	21.0	32.0	31	18.7	25.0	29.6	16.8	23.1
HR (bpm)	100			100			100		
EF_Left_ (%)	27			26.9			31.4		
BSA (m^2^)	1.98			1.98			1.98		
CO (l/min)	2.7			2.7			CO_VENTR_ 0.67		
							Q_VAD_ 2.15		
							TOT 2.82		
CI (l/min/m^2^)	1.36			1.36			0.34		
TPG (mmHg)	10			17			18		
PVR (wood unit)	3.7			6.3			26.87 (18.0/0.67)		
							6.38 (18.0/2.15)		
RVSWI (g/m^2^/beat)	2.4			6.53			6.86		

	Estimated	Simulated	Simulated
EDV (ml)	~ 100	100.4	89.76
ESV (ml)	~ 73	73.4	61.57
Ea (mmHg/ml)	~ 3.0	3.2	2.6
Ees (mmHg/ml)	–	0.88	0.88
Ea/Ees	–	3.64	2.95

*BP* blood pressure, *RA* right atrial pressure, *RV* right ventricular pressure, *PA* pulmonary arterial pressure, *PCWP* pulmonary capillary wedge pressure, *TPG* trans-pulmonary pressure gradient, *CO* cardiac output, *CI* cardiac index, *PVR* pulmonary vascular resistance, *RVSWI* right ventricular stroke work index, *HR* heart rate, BSA body surface area, *EF*_*Left*_ left ventricular ejection fraction, *EDV* end-diastolic volume, *ESV* end-systolic volume, *Ea* arterial elastance, *Ees* slope of end-systolic pressure–volume relationship (ESPVR)

**Fig. 3:**
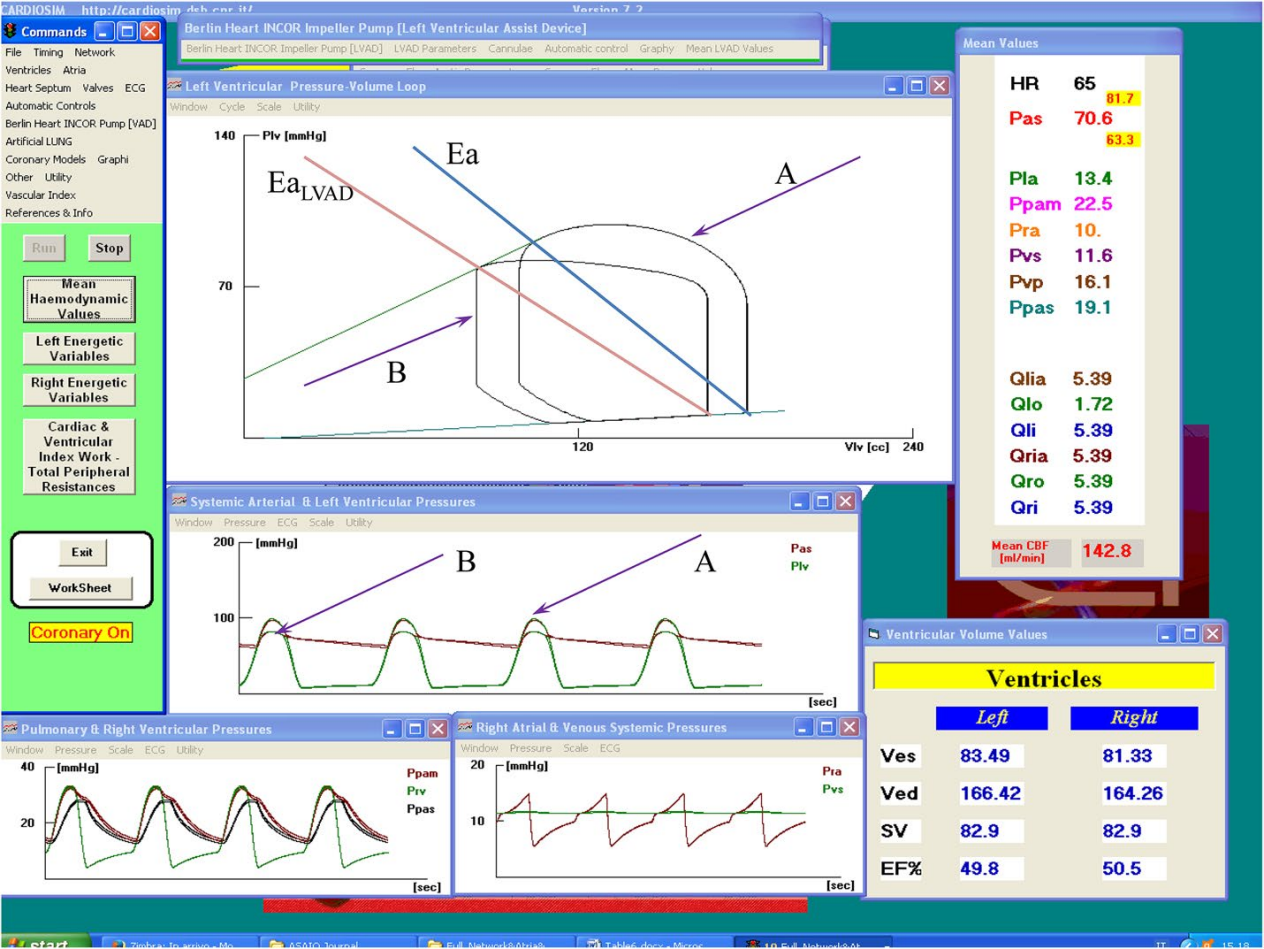
Screen output obtained using our simulator. The first step consists of simulating the “Admission” conditions of the second patient. Subsequently, LVAD assistance is applied. The upper window shows the simulated starting ventricular loop (*A*) and the ventricular loop obtained during LVAD assistance (*B*) in the left ventricular pressure–volume plane. The mean values (calculated during the cardiac cycle in the presence of LVAD assistance) of pressure, flow and HR are reported in the right column. Mean systolic and diastolic values are reported for the systemic arterial pressure (Pas ≡ BP). Pla is the mean left atrial pressure (Pla ≡ PCWP). Pra is the mean right atrial pressure. Mean pulmonary arterial pressure (Ppam ≡ PA), systemic venous pressure (Pvs) and pulmonary venous pressure (Pvp) are also shown. In the bottom column, mean left/right atrial input flow (Qlia/Qria), left/right ventricular input flow (Qli/Qri) and right ventricular output flow (Qro) assume the same value. The sum of the mean left ventricular output flow (Qlo) and the Qvad (LVAD flow) equals the flow into the circulatory network (Qlo + Qvad = Qlia = Qria = Qro = Qri = Qli). Finally, the left lower box reports the end-systolic volume (Ves ≡ ESV), the end-diastolic volume (Ved ≡ EDV), the stroke volume (SV) and the ejection fraction (EF) for both ventricles

Table 6 shows (for the second patient) the comparison between measured (second column) and simulated parameter (third column) for “Admission” conditions. The third and fourth columns represent two different simulated assisted conditions. The first one is obtained applying LVAD support, the second is obtained applying LVAD support and considering the effects induced by Milrinone. Figure 3 shows a screen output produced by our simulator when LVAD support was applied on the simulated “Admission” condition. In the left ventricular pressure–volume plane, the upper window shows the ventricular loop in “Admission” conditions (loop A) and the ventricular loop obtained when LVAD support was applied (loop B). In addition, Fig. 3 shows that the arterial elastance changes as reported in Table 6 where the *Ea/Ees* ratio decreases from 1.62 to 1.32 when LVAD support is applied. When the effects induced by the simultaneous presence of LVAD and pharmacological treatment are simulated, left ventricular ejection fraction EF_Left_ increases from 45.1% (“Admission” conditions) to 55.9% and the *Ea/Ees* ratio decreases from 1.62 (“Admission” conditions) to 1.20. LVAD assistance and Milrinone administration reduce EDV and ESV (Table 6). EDV and ESV reduction induced by LVAD support only is shown in Fig. 3 where a leftward shift of the left ventricular loop is observed.

**Table 6:** Comparison between measured and simulated parameters for patient 2 on admission

Patient #2	Measured (RHC 1)			Simulation (RHC 1)			LVAD (simulation)			LVAD + Mil. (10%) (simulation)		
	Max	Min	Mean	Max	Min	Mean	Max	Min	Mean	Max	Min	Mean
BP (mmHg)	95–100	58	72	97.2	60.5	72	81.7	63.3	70.6	82.2	62.6	70.5
RA (mmHg)	14.0	2.0	9.0	14.9	5.4	10.0	14.9	5.5	10.0	15.2	5.3	10.1
RV (mmHg)	39.0	2.0	17.0	33.5	5.0	14.8	33.6	5.4	15.9	33.4	3.7	14.9
PA (mmHg)	40.0	17.0	27.0	33.0	14.4	23.2	32.2	13.3	22.5	32.9	12.7	22.6
PCWP (mmHg)	28.0	7.0	18.0	26.6	8.9	14.3	25.9	7.9	13.4	27.3	7.6	13.6
HR (bpm)	65			65			65			65		
EF_Left_ (%)	45			45.1			49.8			55.9		
BSA (m^2^)	2.35			2.35			2.35			2.35		
CO (l/min)	5.3			5.3			CO_VENTR_ 1.72			CO_VENTR_ 2.04		
							Q_VAD_ 3.67			Q_VAD_ 3.92		
							TOT 5.39			TOT 5.96		
CI (l/min/m^2^)	2.26			2.26			0.73			0.82		
TPG (mmHg)	9			8.9			9.1			9.0		
PVR (wood unit)	1.7			1.68			5.3 (9.1/1.72)			4.41 (9.0/2.04)		
							2.48 (9.1/3.67)			2.30 (9.0/3.92)		
RVSWI (g/m^2^/beat)	8.5			6.23			6.0			6.63		

	Estimated	Simulated	Simulated	Simulated
EDV (ml)	~ 181	180.74	166.4	163.83
ESV (ml)	~ 99.6	99.2	83.5	72.2
Ea (mmHg/ml)	~ 1.1	1.1	0.9	0.9
Ees (mmHg/ml)	–	0.68	0.68	0.748
Ea/Ees	–	1.62	1.32	1.2

*BP* blood pressure, *RA* right atrial pressure, *RV* right ventricular pressure, *PA* pulmonary arterial pressure, *PCWP* pulmonary capillary wedge pressure, *TPG* trans-pulmonary pressure gradient, *CO* cardiac output, *CI* cardiac index, *PVR* pulmonary vascular resistance, *RVSWI* right ventricular stroke work index, *HR* heart rate, *BSA* body surface area, *EF*_*Left*_ left ventricular ejection fraction, *EDV* end-diastolic volume, *ESV* end-systolic volume, *Ea* arterial elastance, *Ees* slope of end-systolic pressure–volume relationship (ESPVR)

Finally, Table 7 shows the simulation results of the third patient. Again, the *Ea/Ees* ratio decreased when LVAD support and Milrinone administration were simultaneously applied. Table 7 shows that LVAD support produces a leftward shift of the left ventricular loop in the pressure–volume plane reducing EDV and ESV.

**Table 7:** Comparison between measured and simulated parameters for patient 3 on admission

Patient #3	Measured (RHC 1)			Simulation (RHC 1)			LVAD (simulation)			LVAD + Mil. (10%) (simulation)		
	Max	Min	Mean	Max	Min	Mean	Max	Min	Mean	Max	Min	Mean
BP (mmHg)	100	60	73.3	106.7	60	73.4	82.8	66.6	74.1	85.2	67.4	74.6
RA (mmHg)	14	12	9.0	8.8	1.7	4.4	9.0	1.7	4.6	9.2	0.5	4.6
RV (mmHg)	53	5.0	–	33.0	1.0	11.6	31.5	1.0	11.3	32.4	0.5	11.6
PA (mmHg)	58.0	27.0	37.0	32.8	22.6	28.0	31.3	20.4	26.2	32.2	19.9	26.5
PCWP (mmHg)	39.0	29.0	31.0	28.4	12.3	18.0	26.8	9.5	15.4	28.0	8.9	15.5
HR (bpm)	75			75			75			75		
EF_Left_ (%)	15			15			18.9			21.9		
BSA (m^2^)	2.16			2.16			2.16			2.16		
CO (l/min)	4.2			4.2			CO_VENTR_ 0.74			CO_VENTR_ 0.93		
							Q_VAD_ 3.8			Q_VAD_ 4.22		
							TOT 4.54			TOT 5.15		
CI (l/min/m^2^)	1.94			1.94			0.34			0.43		
TPG (mmHg)	6			10.0			10.8			11		
PVR (wood unit)	1.43			2.38			14.6 (10.8/0.74)			11.83 (11/0.93)		
							2.84 (10.8/3.8)			2.61 (11/4.22)		
RVSWI (g/m^2^/beat)	9.87			8.32			8.23			9.47		

	Estimated	Simulated	Simulated	Simulated
EDV (ml)	~ 373	372.53	320.16	313.74
ESV (ml)	~ 317	316.55	259.6	245.0
Ea (mmHg/ml)	~ 1.6	1.9	1.3	1.2
Ees (mmHg/ml)	–	0.4	0.4	0.44
Ea/Ees	–	4.75	3.25	2.73

*BP* blood pressure, *RA* right atrial pressure, *RV* right ventricular pressure, *PA* pulmonary arterial pressure, *PCWP* pulmonary capillary wedge pressure, *TPG* trans-pulmonary pressure gradient, *CO* cardiac output, *CI* cardiac index, *PVR* pulmonary vascular resistance, *RVSWI* right ventricular stroke work index, *HR* heart rate, BSA body surface area, *EF*_*Left*_ left ventricular ejection fraction, *EDV* end-diastolic volume, *ESV* end-systolic volume, *Ea* arterial elastance, *Ees* slope of end-systolic pressure–volume relationship (ESPVR)

## Discussion

Cardiovascular modelling has been very successful in increasing our knowledge of physiological mechanisms where simplified representations of complex biological systems can be used to study their behaviour at different levels [67]. The Cardiac Physiome project is currently the most ambitious and successful application of mathematical and computational modelling aimed to advance our understanding of physiology using a quantitative multi-scale approach [67, 68]. Cardiovascular modelling has now reached the stage where clinical application in the form of patient-specific modelling may become a daily routine in a non distant future. For this approach to become reality, a model must be reliable, reproducible and reduce uncertainty [67, 68]. While effective in the laboratory, almost all the decision support tools have failed when applied to clinical practice [69]. Therefore, it is essential to overcome skepticism by developing a strong, realistic model that can fulfil the expectations. Our approach is focused on modelling and simulation not as a substitute for clinical experience but as an additional tool to guide therapeutic intervention or predict clinical outcome: the clinician will be the ultimate decision maker. The development of a comprehensive, integrated model of the cardiovascular system based on lumped-parameter models, modified time-varying elastance and pressure–volume analysis of ventricular function is an attractive prospect with a view to clinical application. The differential equations describing such a model can be solved relatively easy and yield answers in terms of pressure–volume loops and time-dependent tracings of pressure, flow and volume that may well help the decision process and management in the clinical setting. CARDIOSIM^©^ fulfils these requirements although we acknowledge that other software such as CircAdapt Simulator, HemoLab and Harvi may be potentially suitable for clinical application. The CircAdapt Simulator is based on the CircAdapt model [70–72], which has been designed to simulate the dynamics of the heart and the circulation with the inclusion of a realistic relationship between pressure–volume load and tissue mechanics where the geometry of the components is obtained by adaptation to mechanical load. The implementation of the TriSeg model [73] enables realistic simulation of ventricular mechanics including interactions between left and right ventricle, dynamics of septal geometry and myofibre mechanics in the three ventricular walls. The interesting feature of the CircAdapt model is its combined adaptation of heart and vessels over a relatively long period of time resulting in self-structuring of the circulation as a system where a steady-state solution is obtained [74, 75]. This feature makes the model a potential tool for clinical application with the aim of predicting the evolution of a diseased status and the effects of an interventional procedure [67, 71, 72, 76, 77] but there is a lack of suitable models for VADs support. Despite its limitations, the CircAdapt model is considered easy to use, requires relatively low computational time and allows realistic simulations of the circulation with boundary conditions suitable for more complicated models based on finite element analysis. HeMoLab (Haemodynamics Modelling Laboratory) is an integrated computational environment for the modelling of the cardiovascular system. It is an effective research tool and a virtual simulation laboratory [78–80]. HeMoLab consists of a combination of models, which can be coupled locally and globally in order to obtain the systemic response of the cardiovascular system: the so called 3-D, 1-D and 0-D models. The propagation of the arterial pulse is represented with a 1-D and 0-D model, which describes the behaviour of the flow rate, mean pressure and cross-sectional area as a function of time. HeMoLab is a suitable environment for the simulation of the effects of aging, vasodilatation, vasoconstriction, rest and exercise and calculation of characteristic impedance of the arterial network. Although attractive, the software remains confined to a research environment at present. Harvi is an interactive simulation textbook of cardiovascular physiology and haemodynamics based on a previously described electrical circuit [81–83] where ventricular and atrial contraction are represented by a modified time-varying elastance approach [12].

A simulation-based approach as a potential preoperative strategy in the context of patient-specific modelling in advanced heart failure may be an additional tool to obtain accurate predictions of device performance in a clinical setting with treatment optimisation of this complex and challenging group of patients [21]. Patients in advanced heart failure are critical and their management can be very demanding with emotion running high when dealing with younger patients. Patient 1 is a typical example of difficult case experienced by one of the authors (MC). Co-morbidities do play a role and must be taken into account during the multidisciplinary team meeting. The clinical cases considered are typical heart failure patients referred for assessment for transplant or LVAD insertion. The outcome of the simulations is quite stimulating and open to debate.

The simulation outcome for patient 1 does show a leftward shift of the pressure–volume loops for the left and right ventricles with adequate ventricular volumes, although the gain in ejection fraction may be initially limited. On the contrary, it is true that further improvement is observed over a period of time [84] making LVAD insertion a suitable option for this patient. Because of the potential complications related to the associated co-morbidities, the unanimous clinical decision to decline any further intervention but continue with medical treatment and eventually palliation may have been the way forward although further argument in favour of LVAD insertion would be the recent evidence supporting the “obesity paradox” in cardiac surgery [85], which is currently the subject of a hot debate.

Simulation of the baseline haemodynamic status for patient 2 shows comparable mean values with those measured during right heart catheter. VAD support shows improved left ventricular ejection fraction with some reduction in pulmonary artery pressures. The outcome for patient 3 is most beneficial with significant reduction of pulmonary artery pressures confirming the primary indication for VAD support to achieve pulmonary haemodynamics compatible with transplantation.

In view of its features, CARDIOSIM^©^ may be a more suitable choice with particular reference to the effects of mechanical circulatory support on the cardiovascular system. Lumped-parameter models assume a uniform distribution of pressure, volume and flow within any specific compartment at any instant in time while higher dimensional models recognise the variation in space of these parameters. Lumped-parameter models consist of simultaneous ordinary differential equations complemented by an algebraic balance equation and are suitable for examination of global distribution of pressure, flow and volume over a range of physiological conditions with inclusion of the interaction between modelled components. Higher dimensional models consist of partial differential equations complemented by balance equations. 1-D models represent wave transmission effects within the vascular system but 3-D numerical solutions are required for complex flow patterns with analytical solutions obtained only for the simplest geometry [86]. A single-branch multiple compartment model of the vascular system is suitable for the evaluation of short-term VAD support compared to an overly detailed vessel branch model where parameter setting becomes quite difficult. Higher level lumped-parameter modelling is required to address the interaction between the circulation and other systems but a compromise between complexity and ability to set the required parameters is needed to personalise an integrated lumped model for a patient-specific approach.

CARDIOSIM^©^ does address the systems interaction with its modular approach and assembly of models with varying degree of complexity although 0-D and 1-D coupling may be required for the evaluation of long-term VAD support.

During our simulations, we have also considered the effect on ventricular–arterial coupling following LVAD support. Age-related vascular stiffening and the concomitant changes in left ventricular diastolic compliance are frequently observed in heart failure with preserved ejection fraction and in aortic valve stenosis [87]. Ventricular–arterial coupling is strictly related to cardiovascular performance and can be accurately quantified in terms of pressure and volume. The left ventricle and the arterial system are considered elastic chambers with known volume elastances where left ventricular performance is measured by *Ees*, which is the slope of the ESPVR while the arterial system is measured by its elastance *Ea*, which is the slope of the arterial end-systolic pressure–stroke volume–effective arterial elastance relationship [65, 87]. The *Ea/Ees* ratio may be considered as a reliable performance index for ventricular–arterial coupling. An *Ea/Ees* ratio close to 1 relates to appropriate coupling between the left ventricle and the arterial system. Impaired coupling occurs in heart failure where *Ees* decreases following left ventricular systolic dysfunction and *Ea* is increased because of elevated impedance and reduced compliance [87]. Our simulations show that LVAD assistance can achieve appropriate ventricular–arterial coupling with an *Ea/Ees* ratio close to 1. The potential of this parameter may well justify further study for its routine clinical application in heart failure patients.

In summary, a simulation setting may well add a more quantitative approach to help the whole process, generate more critical thinking and perhaps give reassurance. The clinical scenarios discussed in this article are only an example of how the subject can be further developed and used as part of a preoperative planning strategy. Further work is currently being undertaken by our group and the outcome is awaited.

## Conclusion

Although previous experience, co-morbidities and the risk of potentially fatal complications play a role in the clinical decision process, a simulation setting may well add a more quantitative approach and perhaps reassurance even if the clinician remains the ultimate decision-maker. Interactive software like CARDIOSIM^©^ can reproduce physiological and pathological conditions for clinical decision-making in a controlled environment. Its modular approach and versatility combined with the high availability of physiological and pathological set up make it suitable for clinical application with particular reference to the evaluation of the effects of interventional procedures on the cardiovascular system such as the interactions with pulsatile and continuous flow VADs and the intra-aortic balloon pump (IABP).

The development of an integrated model of the cardiovascular system based on lumped-parameter representation, modified time-varying elastance and pressure–volume analysis of ventricular function seems a feasible and suitable approach yielding a sufficiently accurate quantitative analysis in real time. The challenge remains the ability to predict outcome over a longer period of time.

Patient-specific modelling may become a daily approach for clinical management and optimisation of device treatment. Willingness to adopt such an integrated approach may be the key to further progress.

## Abbreviations

$\varepsilon_{0}$: phase difference between the lvad pulsation component and the native cardiac timing
$A_{0}$: component of the LVAD speed
$A_{\text{p}}$: amplitude of the pulsation component of the LVAD
BMI: body mass index (kg/m^2^)
BP: blood pressure (mmHg)
BSA: body surface area (m^2^)
CFD: computational fluid dynamics
$C_{AT}$: aortic tract compliance (ml/mmHg)
$C_{ABT}$: abdominal tract compliance (ml/mmHg)
CI: cardiac index (l/min/m^2^)
CO: cardiac output (l/min)
CO_VENTR_: left ventricular output flow (l/min)
$C_{pam}$: main pulmonary section compliance (ml/mmHg)
$C_{pas}$: small pulmonary section compliance (ml/mmHg)
$C_{TT}$: thoracic tract compliance (ml/mmHg)
$C_{vp}$: pulmonary venous compliance (ml/mmHg)
$C_{vpi}$: inlet pump cannula compliance (ml/mmHg)
$C_{vpo}$: outlet pump cannula compliance (ml/mmHg)
$C_{vs}$: venous section compliance (ml/mmHg)
E/A wave ratio: the ratio of peak velocity flow in early diastole (the E wave) to peak velocity flow in late diastole caused by atrial contraction (the A wave)
*Ea*: arterial elastance (mmHg/ml)
$e_{Asp}$: septal atrial elastance (mmHg/ml)
ECG: electrocardiogram
EDV: end-diastolic volume (ml)
*Ees*: end-systolic elastance (mmHg/ml)
EF: ejection fraction
EF_Left_: left ventricular ejection fraction
$e_{la}$: left atrial elastance (mmHg/ml)
$e_{lv}$: left ventriclular elastance (mmHg/ml)
$\overline{e}_{lv}$: left ventricular activation function
$E_{lv,d}$: diastolic left ventricular elastance (mmHg/ml)
$E_{lv,s}$: systolic left ventricular elastance (mmHg/ml)
$e_{ra}$: right atrial elastance (mmHg/ml)
$e_{rv}$: right ventricular elastance (mmHg/ml)
$\overline{e}_{rv}$: right ventricular activation function
$E_{rv,d}$: diastolic right ventricular elastance (mmHg/ml)
$E_{rv,s}$: systolic right ventricular elastance (mmHg/ml)
ESV: end-systolic volume (ml)
$e_{Vsp} \left( t \right)$: septal ventricular elastance (mmHg/ml)
ESPVR: end-systolic pressure–volume relationship
HR: heart rate (bpm)
IABP: intra-aortic balloon pump
IAS: interatrial septum
ICD: implantable cardioverter defibrillator
IVS: interventricular septum
$$ K_{{{\text{vad}},{\text{i }}}} \left( {i = 1,2, \ldots ,6} \right) $$: $$ Q_{vad} $$ parameters
$L_{AT}$: aortic tract inertance (mmHg s^2^/ml)
$L_{ABT}$: abdominal tract inertance (mmHg s^2^/ml)
$L_{pam}$: main pulmonary section inertance (mmHg s^2^/ml)
$L_{pas}$: small pulmonary section inertance (mmHg s^2^/ml)
$L_{TT}$: thoracic tract inertance (mmHg s^2^/ml)
LVAD: left ventricular assist device
$L_{vpi}$: inlet pump cannula inertance (mmHg s^2^/ml)
$L_{vpo}$: outlet pump cannula inertance (mmHg s^2^/ml)
MDT: multidisciplinary team
PA: pulmonary arterial pressure (mmHg)
$P_{as}$: systemic arterial pressure (mmHg)
$Pas_{ABT}$: abdominal tract systemic arterial pressure (mmHg)
$Pas_{TT}$: thoracic tract systemic arterial pressure (mmHg)
PCWP: pulmonary capillary wedge pressure (mmHg)
$P_{la}$: left atrial pressure (mmHg)
$P_{lv}$: left ventricular pressure (mmHg)
$P_{lv, 0}$: resting left ventricular pressure (mmHg)
Ppam: mean pulmonary arterial pressure (mmHg)
$P_{ra}$: right atrial pressure (mmHg)
$P_{rv}$: right ventricular pressure (mmHg)
$P_{rv, 0}$: resting right ventricular pressure (mmHg)
$P_{t}$: mean intrathoracic pressure (mmHg)
$P_{vpi}$: inlet cannula pressure (mmHg)
$P_{vpo}$: outlet cannula pressure (mmHg)
$P_{vs}$: systemic venous pressure (mmHg)
PVR: pulmonary vascular resistance (mmHg s/ml)
$Q_{as}$: systemic arterial flow (l/min)
$Q_{AT}$: aortic tract flow (l/min)
$Q_{ABT}$: abdominal tract flow (l/min)
Qli: mean left ventricular input flow (l/min)
Qlia: mean left atrial input flow (l/min)
Qlo: mean left ventricular output flow (l/min)
Qri: mean right ventricular input flow (l/min)
Qria: mean right atrial input flow (l/min)
Qro: mean right ventricular output flow (l/min)
$Q_{TT}$: thoracic tract flow (l/min)
$Q_{vad}$: LVAD flow (l/min)
$Q_{vpi}$: inlet LVAD cannula flow (l/min)
$Q_{vpo}$: outlet LVAD cannula flow (l/min)
RA: right atrial pressure (mmHg)
$R_{AT}$: aortic tract resistance (mmHg s/ml)
$R_{ABT}$: abdominal tract (mmHg s/ml)
$R_{as}$: systemic peripheral resistance (mmHg s/ml)
RHC: right heart catheterisation
RLC: resistor, inductor, capacitor
$R_{pam}$: main pulmonary section resistance (mmHg s/ml)
$R_{par}$: arteriole resistance (mmHg s/ml)
$R_{pas}$: small pulmonary section resistance (mmHg s/ml)
$R_{pc}$: capillary resistance (mmHg s/ml)
$R_{TT}$: thoracic tract resistance (mmHg s/ml)
RV: right ventricle pressure (mmHg)
$R_{vp}$: pulmonary venous resistance (mmHg s/ml)
$R_{vpi}$: inlet pump cannula resistance (mmHg s/ml)
$R_{vpo}$: outlet pump cannula resistance (mmHg s/ml)
$R_{vs1}$: venous system resistance (mmHg s/ml)
$R_{vs2}$: venous system resistance (mmHg s/ml)
RVSWI: right ventricular stroke work index (g/m^2^/beat)
SV: stroke volume (ml)
$T$: duration of the ECG signal (s)
TPG: trans-pulmonary pressure gradient (mmHg)
$T_{TE}$: end of ventricular systole in the ECG signal (s)
$T_{T}$: t-wave peak time in the ECG signal (s)
VAD: ventricular assist device
Ved: end-diastolic volume (ml)
Ves: end-systolic volume (ml)
$V_{la} \left( t \right)$: left atrial volume (ml)
$V_{la, 0}$: resting left atrial volume (ml)
$V_{lv} \left( t \right)$: left ventricular volume (ml)
$V_{lv, 0}$: resting left ventricular volume (ml)
$V_{lv}^{*} \left( t \right)$: left ventricular free wall volume (ml)
$V_{ra} \left( t \right)$: right atrial volume (ml)
$V_{ra, 0}$: resting right atrial volume (ml)
$V_{rv} \left( t \right)$: right ventricular volume (ml)
$V_{rv, 0}$: resting right ventricular volume (ml)
$V_{rv}^{*} \left( t \right)$: right ventricular free wall volume (ml)

## References

[1] Pourmehran O, Rahimi-Gorji M, Gorji-Bandpy M, Gorji TB (2015). Simulation of magnetic drug targeting through tracheobronchial airways in the presence of an external non-uniform magnetic field using Lagrangian magnetic particle tracking. J Magn Magn Mater.

[2] Pourmehran O, Gorji TB, Gorji-Bandpy M (2016). Magnetic drug targeting through a realistic model of human tracheobronchial airways using computational fluid and particle dynamics. Biomech Model Mechanobiol.

[3] Pourmehran O, Rahimi-Gorji M, Ganji DD (2017). Analysis of nanofluid flow in a porous media rotating system between two permeable sheets considering thermophoretic and Brownian motion. Thermal Sci.

[4] Rahimi-Gorji M, Pourmehran O, Gorji-Bandpy M, Gorji TB (2015). CFD simulation of airflow behaviour and particle transport and deposition in different breathing conditions through the realistic model of human airways. J Mol Liquids.

[5] Yousefi M, Pourmehran O, Gorji-Bandpy M, Inthavong K, Yeo L, Tu J (2017). CFD simulation of aerosol delivery to a human lung via surface acoustic wave nebulisation. Biomech Model Mechanobiol.

[6] Tabassum R, Mehmood R, Pourmehran O, Akbar NS, Gorji-Bandpy M (2017). Impact of viscosity variation on oblique flow of Cu–H_2_O nanofluid. Proc I Mech Eng Part E.

[7] Wong KKL, Kelso RM, Worthley SG, Sanders P, Mazumdar J, Abbott D (2009). Cardiac flow analysis applied to phase contrast magnetic resonance imaging of the heart. Ann Biomed Eng.

[8] Wong KK, Tu J, Kelso RM, Worthley SG, Sanders P, Mazumdar J, Abbott D (2010). Cardiac flow component analysis. Med Eng Phys..

[9] Wong KKL, Wang D, Ko JKL, Mazumdar J, Le T-T, Ghista D (2017). Computational medical imaging and hemodynamics framework for functional analysis and assessment of cardiovascular structures. BioMed Eng OnLine.

[10] Wang D, Fong S, Wong RK, Mohammed S, Fiaidhi J, Wong KKL (2017). Robust high-dimensional bioinformatics data streams mining by ODR-ioVFDT. Sci Rep.

[11] Li J, Fong S, Wong RK, Millham R, Wong KKL (2017). Elitist binary wolf search algorithm for heuristic feature selection in high-dimensional bioinformatics datasets. Sci Rep.

[12] Doshi D, Burkhoff D (2016). Cardiovascular simulation of heart failure. Pathophysiol Ther J Card Fail.

[13] Ferrari G, Di Molfetta A, Zieliński K, Fresiello L (2015). Circulatory modelling as a clinical decision support and an educational tool. Biomed Data J.

[14] De Lazzari C, Genuini I, Pisanelli DM, D’Ambrosi A, Fedele F (2014). Interactive simulator for e-Learning environments: a teaching software for health care professionals. BioMed Eng OnLine.

[15] De Lazzari C, Pirckhalava M (2017). Cardiovascular and pulmonary artificial organs: educational training simulators.

[16] Ferrari G, De Lazzari C, Mimmo R, Tosti G, Ambrosi D (1992). A modular numerical model of the cardiovascular system for studying and training in the field of cardiovascular physiopathology. J Biomed Eng.

[17] De Lazzari C, Darowski M, Wolski P, Ferrari G, Tosti G (2005). In vivo and simulation study of artificial ventilation effects on energetic variables in cardiosurgical patients. Methods Inf Med.

[18] De Lazzari C, Quatember B (2016). Cardiac energetics in presence of lung assist devices: in silico study. Model Num Sim Mater Sci.

[19] De Lazzari C (2012). Interaction between the septum and the left (right) ventricular free wall in order to evaluate the effects on coronary blood flow: numerical simulation. Comput Methods Biomech Biomed Eng.

[20] Kozarski M, Ferrari G, Zieliński K, Górczyńska K, Palko KJ, Tokarz A, Darowski M (2008). Open loop hybrid circulatory model: the effect of the arterial lumped parameter loading structure on selected ventricular and circulatory variables. Biocybernet Biomed Eng.

[21] Fresiello L, Ferrari G, Di Molfetta A, Zieliński K, Tzallas A, Jacobs S, Darowski M, Kozarski M, Meyns B, Katertsidis NS, Karvounis EC, Tsipouras MG, Trivella MG (2015). A cardiovascular simulator tailored for training and clinical uses. J Biomed Inform.

[22] De Lazzari C, Darowski M, Ferrari G, Pisanelli DM, Tosti G (2006). The impact of rotary blood pump in conjunction with mechanical ventilation on ventricular energetic parameters: numerical simulation. Methods Inf Med.

[23] De Lazzari C, Darowski M, Ferrari G, Pisanelli DM, Tosti G (2006). Modelling in the study of interaction of hemopump device and artificial ventilation. Comput Biol Med.

[24] De Lazzari C, Darowski M, Ferrari G, Clemente F (1998). The influence of left ventricle assist device and ventilatory support on energy-related cardiovascular variables. Medical Eng Phys.

[25] Shu F, Vandenberghe S, Antaki JF (2009). The importance of dQ/dt on the flow field in a turbodynamic pump with pulsatile flow. Artif Organs.

[26] Bazilevs Y, Gohean JR, Hughes TJR, Moser RD, Zhang Y (2009). Patient-specific isogeometric fluid-structure interaction analysis of thoracic aortic blood flow due to implantation of the Jarvik 2000 left ventricular assist device. Comput Methods Appl Mech Eng.

[27] Peiró J, Veneziani A, Formaggia L, Quarteroni A, Veneziani A (2009). Reduced models of the cardiovascular system. Cardiovascular mathematics. Modelling and simulation of the cardiovascular system.

[28] Suga H, Sagawa K (1972). Mathematical interrelationship between instantaneous ventricular pressure–volume ratio and myocardial force–velocity relation. Ann Biomed Eng.

[29] Suga H, Sagawa K, Shoukas AA (1973). Load independence of the instantaneous pressure–volume ratio of the canine left ventricle and effects of epinephrine and heart rate on the ratio. Circ Res.

[30] Suga H, Sagawa K (1974). Instantaneous pressure–volume relationships and their ratio in the excised, supported canine left ventricle. Circ Res..

[31] Claessens TE, Georgakopoulos D, Afanasyeva M, Vermeersch SJ, Millar HD, Stergiopulos N, Westerhof N, Verdonck PR, Segers P (2006). Nonlinear isochrones in murine left ventricular pressure–volume loops: how well does the time-varying elastance concept hold?. Am J Physiol Heart Circ Physiol.

[32] Vandenberghe S, Segers P, Steendijk P, Meyns B, Dion RAE, Antaki JF, Verdonck P (2006). Modelling ventricular function during cardiac assist: does time-varying elastance work?. ASAIO J.

[33] Stergiopulos N, Meister JJ, Westerhof N (1996). Determinants of stroke volume and systolic and diastolic aortic pressure. Am J Physiol Heart Circ Physiol.

[34] Segers P, Stergiopulos N, Westerhof N (2000). Quantification of the contribution of cardiac and arterial remodeling to hypertension. Hypertension.

[35] Segers P, Steendijk P, Stergiopulos N, Westerhof N (2001). Predicting systolic and diastolic aortic pressure and stroke volume in the intact sheep. J Biomech.

[36] Lankhaar JW, Rövekamp FA, Steendijk P, Faes TJC, Westerhof BE, Kind T, Vonk-Noordegraaf A, Westerhof N (2009). Modeling the instantaneous pressure–volume relation of the left ventricle: a comparison of six models. Ann Biomed Eng.

[37] Pironet A, Desaive T, Kosta S, Lucas A, Paeme S, Collet A, Pretty CG, Kolh P, Dauby PC (2013). A multi-scale cardiovascular system model can account for the load-dependence of the end-systolic pressure–volume relationship. Biomed Eng OnLine.

[38] Negroni JA, Lascano EC (1999). Concentration and elongation of attached cross-bridges as pressure determinants in a ventricular model. J Mol Cell Cardiol.

[39] Smith BW, Chase JG, Nokes RI, Shaw GM, David T (2003). Velocity profile method for time varying resistance in minimal cardiovascular system models. Phys Med Biol.

[40] Smith BW, Chase JG, Nokes RI, Shaw GM, Wake G (2004). Minimal haemodynamic system model including ventricular interaction and valve dynamics. Med Eng Phys.

[41] Luo C, Ramachandran D, Ware DL, Ma TS, Clark JW (2011). Modelling left ventricular diastolic dysfunction: classification and key indicators. Theor Biol Med Model.

[42] Luo C, Ware DL, Zwischenberger JB, Clark JW (2008). A mechanical model of the human heart relating septal function to myocardial work and energy. Cardiovasc Eng.

[43] Olansen JB, Clark JW, Khoury D, Ghorbel F, Bidani A (2000). A closed-loop model of the canine cardiovascular system that includes ventricular interaction. Comput Biomed Res.

[44] Chung DC, Niranjan SC, Clark JW, Bidani A, Johnston WE, Zwischenberger JB, Traber DL (1997). A dynamic model of ventricular interaction and pericardial influence. Am J Physiol Heart Circ Physiol.

[45] Ursino M (1998). Interaction between carotid baroregulation and the pulsating heart: a mathematical model. Am J Physiol Heart Circ Physiol.

[46] Wang Y, Loghmanpour N, Vandenberghe S, Ferreira A, Keller B, Gorcsan J, Antaki J (2014). Simulation of dilated heart failure with continuous flow circulatory support. PLoS ONE.

[47] Huang H, Yang M, Wu S, Liao H (2013). Dynamic modelling of the outlet of a pulsatile pump incorporating a flow-dependent resistance. Med Eng Phys.

[48] Gohean JR, George MJ, Pate TD, Kurusz M, Longoria RG, Smalling RW (2013). Verification of a computational cardiovascular system model comparing the hemodynamics of a continuous flow to a synchronous valveless pulsatile flow left ventricular assist device. ASAIO J.

[49] Nordsletten D, Kay D, Smith N (2010). A non-conforming monolithic finite element method for problems of coupled mechanics. J Comput Phys.

[50] McCormick M, Nordsletten D, Kay D, Smith N (2011). Modelling left ventricular function under assist device support. Int J Numer Methods Biomed Eng.

[51] Baaijens FP (2001). A fictitious domain/mortar element method for fluid–structure interaction. Int J Numer Meth Fluids.

[52] van Loon R, Anderson PD, van de Vosse FN (2006). A fluid–structure interaction method with solid-rigid contact for heart valve dynamics. J Comput Phys.

[53] Korakianitis T, Shi Y (2006). Numerical simulation of cardiovascular dynamics with healthy and diseased heart valves. J Biomech.

[54] Korakianitis T, Shi Y (2006). A concentrated parameter model for the human cardiovascular system including heart valve dynamics and atrioventricular interaction. Med Eng Phys.

[55] Shi Y, Brown AG, Lawford PV, Arndt A, Nuesser P, Hose DR (2011). Computational modelling and evaluation of cardiovascular response under pulsatile impeller pump support. Interface Focus.

[56] Shi Y, Korakianitis T, Bowles C (2007). Numerical simulation of cardiovascular dynamics with different types of VAD assistance. J Biomech.

[57] Shi Y, Korakianitis T (2006). Numerical simulation of cardiovascular dynamics with left heart failure and in-series pulsatile ventricular assist device. Artif Organs.

[58] McCormick M, Nordsletten DA, Kay D, Smith NP (2013). Simulating left ventricular fluid-solid mechanics through the cardiac cycle under LVAD support. J Comput Phys.

[59] Truby L, Naka Y, Bindu K, Ota T, Kirtane AJ, Kodali S, Nikic N, Mundy L, Colombo P, Jorde UP, Takayama H (2015). Important role of mechanical circulatory support in acute myocardial infarction complicated by cardiogenic shock. Eur J Cardiothorac Surg..

[60] De Lazzari C, Stalteri D. CARDIOSIM cardiovascular software simulator. http://cardiosim.dsb.cnr.it/.

[61] Darowski M, De Lazzari C, Ferrari G, Clemente F, Guaragno M (1999). The influence of simultaneous intra-aortic balloon pumping and mechanical ventilation on hemodynamic parameters—numerical simulation. Front Med Biol Eng.

[62] De Lazzari C, Darowski M, Ferrari G, Clemente F, Guaragno M (2001). Ventricular energetics during mechanical ventilation and intra-aortic balloon pumping-computer simulation. J Med Eng Technol.

[63] De Lazzari C, D’Ambrosi A, Tufano F, Fresiello L (2010). Cardiac resynchronization therapy: could a numericalsimulator be a useful tool in order to predict the response of the biventricular pacemaker synchronization?. Eur Rev Med Pharmacol Sci.

[64] Maughan WL, Sunagawa K, Sagawa K (1987). Ventricular systolic interdependence: volume elastance model in isolated canine hearts. Am J Physiol Heart Circ Physiol.

[65] Kelly RP, Ting CT, Yang TM, Liu CP, Maughan WL, Chang MS, Kass DA (1992). Effective arterial elastance as index of arterial vascular load in humans. Circulation.

[66] De Lazzari C, Neglia D, Ferrari G, Bernini F, Micalizzi M, L’Abbate A, Trivella MG (2009). Computer simulation of coronary flow waveforms during caval occlusion. Methods Inf Med.

[67] Lumens J, Leenders GE, Cramer MJ, De Boeck BW, Doevendans PA, Prinzen FW, Delhaas T (2012). Mechanistic evaluation of echocardiographic dyssynchrony indices: patient data combined with multiscale computer simulations. Circ Cardiovasc Imaging.

[68] Bassingthwaighte J, Hunter P, Noble D (2009). The cardiac physiome: perspectives for the future. Exp Physiol.

[69] Yang Q, Zimmerman J, Steinfeld A, Carey L, Antaki JF (2016). Investigating the heart pump implant decision process: opportunities for decision support tools to help. ACM Trans Comput Hum Interact.

[70] Arts T, Delhaas T, Bovendeerd P, Verbeek X, Prinzen FW (2005). Adaptation to mechanical load determines shape and properties of heart and circulation: the circadapt model. Am J Physiol Heart Circ Physiol.

[71] Lumens J, Delhaas T (2012). Cardiovascular modeling in pulmonary arterial hypertension: focus on mechanisms and treatment of right heart failure using the circadapt model. Am J Cardiol.

[72] Lumens J (2014). Creating your own virtual patient with circadapt simulator. Eur Heart J.

[73] Lumens J, Delhaas T, Kirn B, Arts T (2009). Three-wall segment (triseg) model describing mechanics and hemodynamics of ventricular interaction. Ann Biomed Eng.

[74] Arts T, Lumens J, Kroon W, Delhaas T (2012). Control of whole heart geometry by intramyocardial mechano-feedback: a model study. PLoS Comput Biol.

[75] Arts T, Reesink K, Kroon W, Delhaas T (2011). Simulation of adaptation of blood vessel geometry to flow and pressure: implications for arterio-venous impedance. Mech Res Commun.

[76] Lumens J, Arts T, Marcus JT, Vonk-Noordegraaf A, Delhaas T (2012). Early-diastolic left ventricular lengthening implies pulmonary hypertension-induced right ventricular decompensation. Cardiovasc Res.

[77] Lumens J, Ploux S, Strik M, Gorcsan J, Cochet H, Derval N, Strom M, Ramanathan C, Ritter P, Haissaguerre M, Jais P, Arts T, Delhaas T, Prinzen FW, Bordachar P (2013). Comparative electromechanical and hemodynamic effects of left ventricular and biventricular pacing in dyssynchronous heart failure: electrical resynchronization versus left–right ventricular interaction. J Am Coll Cardiol.

[78] Larrabide I, Blanco PJ, Urquiza SA, Dari EA, Vénere MJ, de Souza e Silva NA, Feijóo RA (2012). HeMoLab—Haemodynamics Modelling Laboratory: an application for modelling the human cardiovascular system. Comput Biol Med.

[79] Blanco PJ, Clausse A, Feijóo RA (2017). Homogenization of the Navier–stokes equations by means of the multi-scale virtual power principle. Comput Methods Appl Mech Eng.

[80] HeMoLab (Hemodynamics Modeling Laboratory) http://hemolab.lncc.br/.

[81] Harvi. Interactive software simulator of cardiovascular physiology http://www.pvloops.com/.

[82] Santamore WP, Burkhoff D (1991). Haemodynamic consequences of ventricular interaction as assessed by model analysis. Am J Physiol Heart Circ Physiol.

[83] Burkhoff D, Tyberg JV (1993). Why does pulmonary venous pressure rise after onset of left ventricular dysfunction: a theoretical analysis. Am J Physiol Heart Circ Physiol.

[84] Capoccia M, Bowles CT, Sabashnikov A, De Robertis F, Amrani M, Banner NR, Simon A (2015). A UK single centre retrospective analysis of the relationship between haemodynamic changes and outcome in patients undergoing prolonged left ventricular assist device support. Ann Thorac Cardiovasc Surg.

[85] Mariscalco G, Wozniak MJ, Dawson AG, Serraino GF, Porter R, Nath M, Klersy C, Kumar T, Murphy GJ (2017). Body mass index and mortality among adults undergoing cardiac surgery. a nationwide study with a systematic review and meta-analysis. Circulation.

[86] Shi Y, Lawford P, Hose R (2011). Review of zero-D and 1-D models of blood flow in the cardiovascular system. BioMed Eng OnLine.

[87] Antonini-Canterin F, Carerj S, Di Bello V, Di Salvo G, La Carrubba S, Vriz O, Pavan D, Balbarini A, Nicolosi GL (2009). Arterial stiffness and ventricular stiffness: a couple of diseases or a coupling disease? A review from the cardiologist’s point of view. Eur J Echocardiogr.

